# Defining the genetic architecture of hypertrophic cardiomyopathy: re-evaluating the role of non-sarcomeric genes

**DOI:** 10.1093/eurheartj/ehw603

**Published:** 2017-01-11

**Authors:** Roddy Walsh, Rachel Buchan, Alicja Wilk, Shibu John, Leanne E Felkin, Kate L Thomson, Tang Hak Chiaw, Calvin Chin Woon Loong, Chee Jian Pua, Claire Raphael, Sanjay Prasad, Paul J Barton, Birgit Funke, Hugh Watkins, James S Ware, Stuart A Cook

**Affiliations:** 1 NIHR Cardiovascular Biomedical Research Unit, Royal Brompton and Harefield NHS Foundation Trust and Imperial College London, Sydney Street, London SW3 6NP, UK; 2 Cardiovascular Genetics and Genomics, National Heart and Lung Institute, Imperial College London, Sydney Street, London SW3 6NP, UK; 3 Oxford Medical Genetics Laboratory, Oxford University Hospitals NHS Foundation Trust, The Churchill Hospital, Old Road, Headington, Oxford OX3 7LE, UK; 4 Radcliffe Department of Medicine, Level 6, West Wing, John Radcliffe Hospital, Headley Way, Headington, Oxford OX3 9DU, UK; 5 National Heart Research Institute Singapore, National Heart Centre Singapore, 5 Hospital Drive, 169609 Singapore, Singapore; 6 NIHR Cardiovascular Biomedical Research Unit, Royal Brompton Hospital, Sydney Street, London SW3 6NP, UK; 7 Laboratory for Molecular Medicine, Partners HealthCare Personalized Medicine, 65 Lansdowne Street, Cambridge, MA 02139, USA; 8 Department of Pathology, Massachusetts General Hospital and Harvard Medical School, 55 Fruit Street, Boston, MA 02114, USA; 9 The Wellcome Trust Centre for Human Genetics, Roosevelt Drive, Oxford OX3 7BN, UK; 10 Cardiovascular Magnetic Resonance Imaging and Genetics, MRC London Institute of Medical Sciences, Imperial College London, Hammersmith Hospital Campus, Du Cane Road, London W12 0NN, UK; 11 Division of Cardiovascular & Metabolic Disorders, Duke-National University of Singapore, 8 College Road, 169857 Singapore, Singapore

**Keywords:** Hypertrophic cardiomyopathy, HCM genetics, Mendelian genetics, ExAC, Rare genetic variation

## Abstract

**Aim:**

Hypertrophic cardiomyopathy (HCM) exhibits genetic heterogeneity that is dominated by variation in eight sarcomeric genes. Genetic variation in a large number of non-sarcomeric genes has also been implicated in HCM but not formally assessed. Here we used very large case and control cohorts to determine the extent to which variation in non-sarcomeric genes contributes to HCM.

**Methods and results:**

We sequenced known and putative HCM genes in a new large prospective HCM cohort (*n* = 804) and analysed data alongside the largest published series of clinically genotyped HCM patients (*n* = 6179), previously published HCM cohorts and reference population samples from the exome aggregation consortium (ExAC, *n* = 60 706) to assess variation in 31 genes implicated in HCM. We found no significant excess of rare (minor allele frequency < 1:10 000 in ExAC) protein-altering variants over controls for most genes tested and conclude that novel variants in these genes are rarely interpretable, even for genes with previous evidence of co-segregation (e.g. *ACTN2*). To provide an aid for variant interpretation, we integrated HCM gene sequence data with aggregated pedigree and functional data and suggest a means of assessing gene pathogenicity in HCM using this evidence.

**Conclusion:**

We show that genetic variation in the majority of non-sarcomeric genes implicated in HCM is not associated with the condition, reinforce the fact that the sarcomeric gene variation is the primary cause of HCM known to date and underscore that the aetiology of HCM is unknown in the majority of patients.

## Translational perspective

This study comprehensively evaluates the role of non-sarcomeric genes that have been implicated in HCM. Most of the genes analysed displayed no significant excess of rare variation in HCM cases compared to population reference samples, indicating they are either wrongly associated with the disease or will yield uninterpretable data in a clinical setting. These findings inform which genes have sufficient evidence to be included on routine HCM clinical genetic testing panels and will reduce the number of uncertain or false positive findings obtained from the clinical testing of wrongly implicated genes.

## Introduction 

Hypertrophic cardiomyopathy (HCM) is a common inherited heart disease, affecting at least 1 in 500 people.^1^ It is defined by the presence of left ventricular hypertrophy (LVH) in the absence of other causal cardiac or systemic conditions. Hypertrophic cardiomyopathy is classically regarded as an autosomal dominant Mendelian disease, though one characterized by variable expressivity and penetrance.^2^ The first gene to be linked to HCM (*MYH7*) was identified in 1989,^3^ with its genetic repertoire expanded throughout the 1990s to include a further seven sarcomeric genes (*MYBPC3*, *TNNT2*, *TPM1*, *MYL2*, *MYL3*, *TNNI3*, *ACTC1*).^4^ Pathogenic variants in these genes were identified though linkage studies in multiple large affected pedigrees and the complete segregation of genetic variant with disease phenotype provided definitive evidence of disease association for these genes. More than one thousand variants in these eight sarcomeric genes have since been linked to HCM and the importance and causation of the sarcomeric genes has been irrefutably established.

Recent advances in DNA sequencing technologies have fuelled intense research into the genetic aetiology of HCM. Over the last 15 years, almost 250 variants in over 40 additional, mainly non-sarcomeric genes have implicated in HCM, mostly identified through candidate gene research studies involving genes with a hypothetical role in HCM, e.g. those encoding Z-disc and calcium signalling proteins. In these studies, variant pathogenicity has often been inferred on the basis of rarity in small control or population cohorts, sometimes supported by functional validation. Segregation evidence has tended to be limited and restricted to small pedigrees.

In recent years, data from projects such as the 1000 Genomes, the Exome Sequencing Project and in particular the Exome Aggregation Consortium^5^ (ExAC) has highlighted that rare protein-altering variation is far more common in the general population than previously thought. As well as demonstrating that many variants reported as pathogenic are present at population frequencies incompatible with dominant, Mendelian variants,^6^ they have revealed that individually rare variants are collectively quite common for particular genes. Older studies did not control for this—though individual variants were confirmed as rare, novel candidate genes were seldom fully sequenced in control groups, leaving studies susceptible to false positive disease associations. Demonstrating an overall excess of rare variants across genes in case cohorts is a valuable technique for validating novel genetic associations with Mendelian diseases and likely to be essential for genes to yield interpretable findings in genetic testing.

The ExAC database of 60 706 samples, derived from several different control and common disease cohorts, provides an accurate and increasingly comprehensive representation of genetic variation in the general population and allows us to reassess previous claims of disease associations for HCM. Recently, we compared the level of rare variation between ExAC reference samples and cardiomyopathy cohorts from two major clinical genetics laboratories, the Oxford Molecular Genetics Laboratory (OMGL) and the Partners Laboratory for Molecular Medicine (LMM), which included 6179 HCM cases sequenced in up to 20 genes.^7^ As expected, we confirmed a significant excess of variants in the eight main sarcomeric genes (and three genes associated with metabolic cardiomyopathies). The limited number of non-sarcomeric genes assessed in the clinical laboratories showed little or no excess burden of rare genetic variation, rendering their role in HCM uncertain.

**Figure 1:**
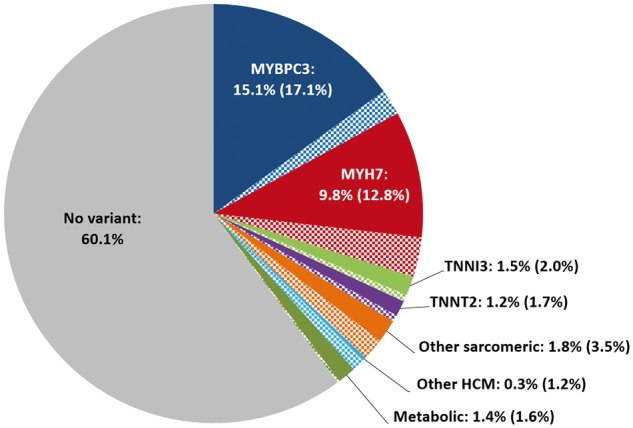
Proportion of HCM patients referred for clinical genetic testing (at OMGL and LMM) with Pathogenic or Likely Pathogenic variants. The percentage in parentheses refer to the total case excess over the frequency of rare variation in ExAC that is expected to reflect the actual yield for each gene (including variants classified as unknown significance shown in the check-patterned slices). The proportions were calculated according to the number of patients sequenced for each gene (up to 6179) and averaged for the three categories - other sarcomeric (ACTC1, MYL2, MYL3, TPM1), other HCM (CSRP3, FHL1, PLN) and metabolic cardiomyopathies (GLA, LAMP2, PRKAG2). Non-essential splice site variants are not included. Over 99% of pathogenic HCM variants occur in the eight core sarcomeric genes (MYH7, MYBPC3, TNNT2, TPM1, MYL2, MYL3, TNNI3, and ACTC1). HCM, Hypertrophic cardiomyopathy.

The Laboratory of Molecular Medicine have recently suggested that expanding gene panels beyond the 11 core sarcomeric and metabolic cardiomyopathy genes offers very little increase in test yield and sensitivity.^8^ Indeed, combining the clinical genetics results from LMM and OMGL confirms that over 99% of variants classed as pathogenic or likely pathogenic by clinical grade criteria for HCM testing are accounted for by the eight core sarcomeric genes (*Figure **1*). It remains to be determined what proportion of the genes recently implicated in HCM are valid disease genes.

To begin to address the role of genetic variation in genes other than the major HCM genes, we sequenced a comprehensive panel of genes previously claimed to be associated with HCM (including over 30 genes not on the standard clinical panels assessed in our previous study)^7^ in a cohort of 804 unrelated HCM cases. We then compared the prevalence of rare genetic variation in cases to that in the ExAC reference population. We re-evaluated published gene and variant links in light of the ExAC data and assessed this cumulative evidence to classify genes as to their likely role in HCM. In light of the trend in clinical and research laboratories towards ever larger gene panels (and whole exome and genome sequencing) in HCM genetic testing, a critical appraisal of the role of genes which have been linked to the disease is essential for the accurate identification of causative variants and to minimize potential false positive and ambiguous results.

## Methods

### Identification of reported hypertrophic cardiomyopathy genes

Genes with variants that have been associated with HCM in published reports were identified by interrogating the Human Gene Mutation Database (HGMD, professional version 2015.3). Entries with a "disease-causing mutation" tag (DM or DM?) with disease descriptions related to HCM were analysed (see Supplementary material online, Supplementary Note 1 for the list of manually curated HCM disease terms and Supplementary material online, *Table S1* for the list of genes analysed). As the primary focus of this study was the genetics of HCM, genes associated with syndromic conditions that include cardiac hypertrophy were not included in our analyses (see Results for details).

### Sequencing of prospective hypertrophic cardiomyopathy cohort

Prospective patients with a diagnosis of HCM, confirmed with reference to established cardiac MRI (UK) and/or echocardiographic (Singapore) diagnostic criteria, underwent comprehensive genetic evaluation. 684, mainly Caucasian, patients were recruited by the Cardiovascular Biomedical Research Unit at the Royal Brompton Hospital, London and 120, mainly Chinese, patients at the National Heart Centre Singapore. For details on the sequencing panels and platforms used and the bioinformatics pipelines used to identify variants, see Supplementary material online, Supplementary Note 2. Supplementary material online, *Table S1* details the number of samples sequenced for each gene and the canonical transcripts used for analysis.

### Exome Aggregation Consortium population reference data

Exome aggregation consortium comprises aggregated sequencing data from a variety of large-scale exome sequencing projects that have been reprocessed through the same pipeline, currently numbering 60 706 samples. Although ExAC is not strictly a control dataset, none of the constituent cohorts are expected to be enriched for inherited cardiac conditions, and we expect the frequency of pathogenic variants to be representative of the general population. Data were downloaded from the ExAC website (http://exac.broadinstitute.org, January 2015) [version 0.3, January 2015]. Rare variants were defined for this study as having a mean allelic frequency (MAF) of less than 1 × 10^−^^4^ in ExAC (as per our previous study based on the ExAC frequency of the most common HCM variant, *MYBPC3*:p.R502W)^7^—this is a conservative cut-off that is not intended to define pathogenicity as such, but rather to ensure that all potentially pathogenic variants are included and to allow for the comparison of rare variant frequencies between different cohorts. Only protein-altering variants (i.e. missense, nonsense, frameshift, inframe indels, and essential splice site) in defined transcripts (see Supplementary material online, *Table S1*) and with high quality calling (PASS filter) were included in the analyses. To minimize any bias resulting from the potentially higher sensitivity sequencing in the HCM gene panel studies, the total number of ExAC samples per gene were adjusted based on the mean coverage at the variant sites of interest.

### Analysis of published data

For the 31 non-core sarcomeric and non-syndromic putative HCM genes, published reports linking variants to HCM were identified from HGMD and Pubmed searches and analysed for data on the frequencies of rare variants in sequenced cohorts, family segregation, *de novo* variation, and functional validation studies. The maximum logarithm of odds (LOD) score for each gene was noted from published scores or estimates from reported pedigrees (only individuals with a HCM/LVH phenotype were included, the presence of the variant in phenotypically-negative individuals was tolerated due to the incomplete penetrance and late disease onset that is characteristic of HCM). At the variant level, individual variants that occurred in multiple cases (> 3) were analysed to assess if they were significantly enriched compared to ExAC (Fisher’s exact test). Variants that were reported to be *de novo* in HCM patients were also noted.

### Genetic data from hypertrophic cardiomyopathy clinical laboratories

We previously analysed genetic data from 6179 unrelated HCM index cases referred for clinical genetic testing at OMGL and LMM, sequenced in up to 12 non-sarcomeric genes.^7^ See Supplementary material online, *Table S1* for number of patients sequenced for each gene and http://cardiodb.org/ACGV/ for further details.

### Comparison of rare variation between hypertrophic cardiomyopathy case cohorts and Exome Aggregation Consortium reference data

For each gene, the frequency of rare variation observed in HCM cases was calculated by dividing the number of rare variants by the total number of cases sequenced. Case frequencies were calculated for the cohort reported in this study, for each previously published cohort and for the combined data from our study, the clinical genetic cohorts and published reports. The case excess was defined by subtracting the rare variant frequency in ExAC from each of the calculated HCM case frequencies. To determine if any observed excess was statistically significant, a Fisher’s exact test was performed with Bonferroni correction for multiple testing (31 genes, corrected significance threshold *P* = 0.0016).

### Classification of hypertrophic cardiomyopathy genes

**Figure 2:**
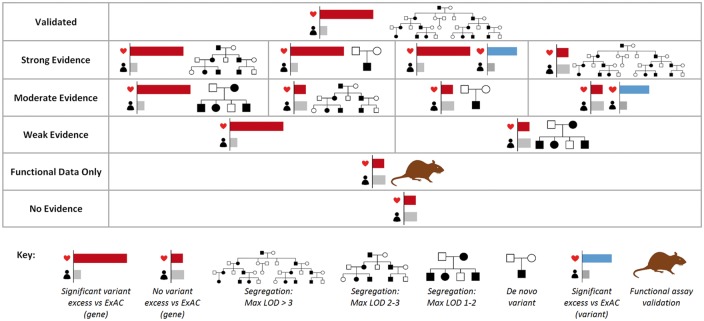
Evidence used to classify genes associated with hypertrophic cardiomyopathy. For full details on the criteria for each category, see Supplementary material online, *Table S2*.

Genes were classified based on the evidence for their association to HCM from several sources—a significant excess of rare variation in the combined case cohorts versus ExAC (across genes or for individual variants), segregation of reported variants, the presence of *de novo* variants in cases, and published studies that assessed the functional effect of variants in cellular, tissue or animal models (*Figure **2* and see Supplementary material online, *Table S2*). Putative causative HCM genes were classified as either validated or having strong, moderate or weak evidence of association. Genes without sufficient human genetic evidence (case excess or segregation) but with functional data associated with rare variants identified in HCM patients were classified as ‘functional data only’. Genes without a significant excess in cases and without segregation/functional data to support published variants were considered to currently have no evidence for association with HCM.

## Results

### Genes associated complex/syndromic conditions including left ventricular hypertrophy

Variants in 51 genes are linked with HCM in the HGMD database, including eight robustly validated sarcomeric HCM genes, 31 non-sarcomeric genes reported as associated with isolated HCM, and 12 associated with complex phenotypes that may include LVH. Of the latter (see Supplementary material online, *Table S3*), five genes are robustly associated with metabolic conditions that can mimic and initially present as HCM and which are regularly included in HCM genetic testing (*LAMP2*, *GAA*, *GLA*, *PRKAG2*, *TTR*). Variants in two ion channel genes (*CACNA1C* and *KCNQ1*) have been identified in patients with mixed HCM and arrhythmia phenotypes but are suggested to be responsible for the arrhythmic rather than hypertrophic component of the phenotype in these cases. Variants in five genes (*ACTA1*, *COX15*, *MRPL3*, *MYO6*, and *SLC25A5*) are reported in other rare and complex disorders which mostly exhibit recessive inheritance patterns. While the metabolic genes should be included in HCM genetic testing, we have not included them in subsequent analysis as they are phenocopies and are not associated with classical, isolated HCM.

### Validated sarcomeric genes

The eight core sarcomeric genes (*MYH7*, *MYBPC3*, *TNNT2*, *TPM1*, *MYL2*, *MYL3*, *TNNI3*, and *ACTC1*) are considered validated due to the detailed family linkage studies that identified them as HCM genes, hundreds of subsequent reports describing further pathogenic variants (many supported by functional and segregation data), the significant excess of rare variation observed in each gene when comparing large clinical cohorts to ExAC reference samples and the detection of multiple pathogenic variants by the clinical grade classifications employed by OMGL and LMM.^7^Supplementary material online, *Table S4* summarizes this evidence.

### Putative hypertrophic cardiomyopathy genes

**Figure 3:**
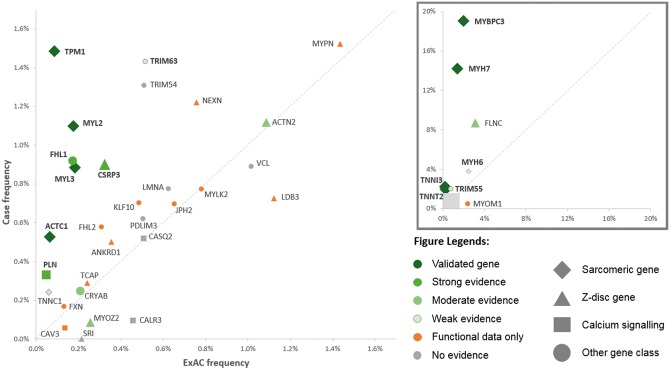
Scatter plot summarizing the evidence for involvement of genes implicated in hypertrophic cardiomyopathy (low frequency genes in main plot, higher frequency genes in subplot). The frequency of rare variants in combined cases is shown on the y-axis and in Exome Aggregation Consortium on the x-axis. Genes with an excess in cases are displayed above the diagonal. Data points are coloured according to classification by this study, shaped according to gene function and sized according to amount of published segregation data.

The 31 putative, mostly non-sarcomeric HCM genes that are the main focus of this study are listed in *Table **1*, along with frequencies of rare variation in ExAC and HCM cohorts and summary of published segregation and functional data. See *Table S5* for more detailed information on each cohort and published study and *Table S7* for the list of rare variants detected in our prospective cohort. The frequency data and published evidence are also summarized in the scatter plot in *Figure **3*.

**Table 1:** **Summary of genetic evidence associating genes to** hypertrophic cardiomyopathy

Gene	Population frequency (ExAC, %)	HCM frequency (%)				Fisher's exact test	Max LOD score	*De novo*	Individual variant excess
		This study	OMGL/LMM clinical data	Published data	Combined data (total cases)				
Strong evidence									
CSRP3	0.32	0.75	0.51	1.42	0.90 (4866)	***	3.6		√
FHL1	0.12	0.49	0.91	2.48	0.92 (2061)	***		√	√
PLN	0.05	0.25	0.32	0.36	0.33 (5440)	***			√
Moderate evidence									
ACTN2	1.09	1.99	0.69	0.93	1.12 (2779)	n/s	2.82		
CRYAB	0.21	0.25			0.25 (804)	n/s		√	
FLNC	3.21			8.70	8.70 (92)	*	2.33		
MYOZ2	0.26	0.12	0.16	0.00	0.08 (2390)	no excess	2.03		
Weak evidence									
MYH6	2.47	3.36		4.20	3.80 (1709)	**			
TNNC1	0.06	0.25	0.00	0.32	0.24 (3335)	*	1.2		
TRIM55	0.73	1.50		2.36	2.01 (993)	***			
TRIM63	0.51	1.00		2.02	1.43 (1398)	***			
Functional data only (no genetic evidence)									
ANKRD1	0.35	0.37	0.50	0.78	0.50 (1995)	n/s			
CAV3	0.14	0.00		0.10	0.06 (1824)	no excess	0.6		
FHL2	0.31	0.50		0.83	0.58 (520)	n/s			
FXN	0.13	0.12		0.26	0.17 (1193)	n/s			
JPH2	0.65	0.99		0.56	0.70 (1292)	n/s			
KLF10	0.49	0.99		0.54	0.68 (1328)	n/s			
LDB3	1.12	1.48		0.45	0.73 (1518)	no excess			
MYLK2	0.78	1.24		0.00	0.77 (1294)	no excess			
MYOM1	2.39			0.53	0.53 (188)	no excess	0.6		
MYPN	1.43	1.49		1.56	1.52 (1381)	n/s			
NEXN	0.76	1.24	1.27	0.83	1.22 (1557)	n/s	0.9		
TCAP	0.24	0.12		0.37	0.29 (2413)	n/s			
No evidence									
CALR3	0.46	0.00		0.40	0.10 (1056)	no excess			
CASQ2	0.51	0.37		0.62	0.52 (1930)	n/s			
LMNA	0.62	1.00		0.57	0.78 (1678)	n/s			
OBSCN	11.07				0.00 (0)				
PDLIM3	0.51	0.87		0.41	0.62 (1771)	n/s			
SRI	0.21			0.00	0.00 (252)	no excess			
TRIM54	0.51	0.25		2.02	1.31 (993)	*			
VCL	1.02	1.00		0.84	0.89 (2358)	no excess			

Summary of rare variant frequencies in putative HCM genes in HCM cases and reference population samples (ExAC). The frequency of rare variants is displayed for ExAC, the prospective HCM cohort sequenced in this study, the clinical sequencing cohorts,^7^ published cohorts and the combined data (this study, clinical sequencing and published data). A Fisher's exact test compared the burden of variation between cases (combined data) and ExAC (n/s = not significant, *= *P* < 0.05, **= *P* < 0.0016 correction for multiple testing threshold, ***= *P* < 0.0001). Maximum LOD scores, reports of *de novo* variants and individual variants with significant excess over ExAC are also noted from published studies. See Supplementary material online, *Table S5* for full details and Supplementary material online, *Table S1* for the number of HCM patients sequenced in this study and in the clinical laboratories.

### Genes with some evidence for a primary pathogenic role in hypertrophic cardiomyopathy

We classified 11 genes as having strong (*CSRP3*, *FHL1*, *PLN*), moderate (*ACTN2*, *CRYAB*, *FLNC*, *MYOZ2*) or weak (*MYH6*, *TNNC1*, *TRIM55*, *TRIM63*) evidence for a primary pathogenic role in HCM based on significant excess of rare variants and supporting human genetics data as outlined in *Figure **2* and Supplementary material online, *Table S2*.

A significant excess of rare variants in the combined HCM cohorts over ExAC was observed in three genes that had additional supporting human genetic evidence, though the excess of variants was less than 1% for each gene. *CSRP3* (Z-disc) had an excess of 0.58%, a maximum LOD score of 3.6 and one variant,^9^ p.L44P, significantly enriched in cases (11/4866 cases versus 0/60706 ExAC samples, *P* < 1 × 10^−^^4^). The X-linked *FHL1* (desmosome) had an excess of 0.80%, a confirmed *de novo* truncating variant^10^ and one variant, p.N38S, significantly enriched in cases (4/2061 cases vs. 0/60706 ExAC samples, *P* < 1 × 10^−^^4^). *PLN* (calcium signalling) had an excess of 0.28% with one variant, p.L39X, significantly enriched in cases (4/5435 cases versus 1/60671 ExAC samples, *P* = 2 × 10^−^^4^). An excess of 0.18% was observed in *TNNC1* (sarcomere) but it fell just below the Bonferroni corrected *P*-value (*P* = 0.0017) due to the rarity of variants in this gene—although it was classified as having weak evidence in this study, further sequencing or segregation data may confirm the gene as a very rare cause of HCM. Although not sequenced here, the Z-disc gene *FLNC* has recently been implicated in HCM,^11^ with strong supporting evidence. However, the high case frequency (in 8/92 probands) was inflated by sequencing only sarcomeric-negative patients and given the relatively high frequency of rare variants in the population (3.2%), more study will be required to characterize the role of *FLNC* in HCM patients.

In contrast, although no excess was observed in Z-disc genes *ACTN2* and *MYOZ2*, variants in these genes have been reported to segregate in relatively large pedigrees,^12–14^ indicating that specific variants may indeed be causative for HCM, though the maximum LOD scores are below the threshold of 3 that typically defines a significant association (ACTN2–2.89, MYOZ2–2.03). While specific variants may be linked with disease in these families, most variants detected in these genes will represent background, non-disease variation (rare variant frequency in ExAC of 1.1% in *ACTN2*, 0.26% in *MYOZ2*). Alternatively, it is possible that while the loci identified in these linkage studies are associated with HCM in these families, the true disease-causing variants are actually in alternative genes at the loci.

Three genes display a significant excess in cases but, in the absence of any other genetic or functional evidence, their role in HCM remains uncertain. In *MYH6* (rare variants detected in 3.8% of cases vs. 2.5% of individuals in ExAC), the high background rate of variants relative to cases makes variant interpretation extremely challenging and renders any finding in a clinical setting essentially unactionable. An excess of variants in *TRIM55* and *TRIM63* have been reported in HCM cases where they were proposed as putative modifiers.^15^ However, the case frequency in our cohort was substantially lower than the published report (1.5% vs 2.4% and 1.0% vs 2.0%, respectively) and not significantly in excess over ExAC.

### Functional data only

A number of genes have been reported as putative novel HCM genes, with rare variants identified in patient cohorts that are supported by functional effects in cellular and animal models. For example, rare variants in the Z-disc gene *MYPN* are detected in 1.5–2.0% of HCM patients, with mice expressing the p.Y20C variant reported to develop a HCM phenotype.^16^ However, this variant is too common to be a primary pathogenic variant (occurring in 111 individuals in ExAC) and the frequency of rare MYPN variants is similar in case and population cohorts. Similarly, several studies have demonstrated effects of variants in *ANKRD1*, *CAV3*, *JPH2*, *KLF10*, *NEXN* and *TCAP* on animal models and various cellular assays (see Supplementary material online, *Table S5*). However, as these genes have no variants with evidence of segregation in families (LOD > 1) or an overall excess of variants in cases, a primary role for them in HCM cannot currently be supported.

### Genes with no convincing evidence of association to hypertrophic cardiomyopathy

For eight genes, there is currently no evidence that the variants that have been associated with HCM have any clinical effect and are more likely to represent background variation. These include genes where reported variants are too common in ExAC (MAF > 1 × 10^−^^4^) to be pathogenic (*LMNA*, *OBSCN*, *SRI*), genes with rare variants frequencies in HCM cohorts similar to ExAC (*CALR3*, *CASQ2*) and a gene (*VCL*) where the functional effect observed in patients with variants (a marked reduction in vinculin protein levels observed in intercalated discs in the patients’ myectomy samples^17^) appears to be a general feature of HCM regardless of genotype.^18^

### Clinical characteristics by genotype status

Patients with rare variants in valid HCM genes were significantly younger, had a greater family history of both HCM and sudden cardiac death and larger maximum LV wall thickness than both genotype negative patients and patients with rare variants in other analysed genes (see Supplementary material online, *Table S6*).

## Discussion

Since the initial linkage studies of the 1990s implicated sarcomeric genes in HCM, years of genetic research have reported over 40 additional putative gene associations. However, these findings have had little impact of clinical genetic testing for HCM, with over 99% of pathogenic variants occurring in established sarcomeric genes and two-thirds of referrals for genetic testing lacking a positive genetic result. To robustly address the apparent paradox of many more genes implicated but very little increase in overall genetic burden explained, we sequenced 804 HCM patients across a comprehensive panel of putative HCM genes, compared the observed variation with a systematic assessment of background population variation and reassessed published data in light of new insights offered by ExAC.

Our findings demonstrate why sequencing of non-sarcomeric genes has not improved the yield of HCM genetic testing. An excess of variants was observed in only four genes (*CSRP3*, *FHL1*, *PLN*, *TNNC1*) that are plausibly pathogenic for HCM, with these genes estimated to yield disease-causing variants in less than 2% of cases overall. *FLNC* is likely a bona fide HCM gene, though the prevalence of pathogenic variants remains uncertain. For the other 26 putative HCM genes, there is little or no excess rare variation over ExAC, indicating they are either very rarely causative for HCM (with the high background variation rendering novel variants uninterpretable in the absence of extensive functional or segregation data) or that the associations were erroneous, based on candidate gene studies that did not appropriately control for the background rare variation rate in the population.

The lack of a significant case excess does not necessarily exclude a role for a gene in Mendelian diseases like HCM. Putative pathogenic variants have been identified through linkage analysis and/or segregation in relatively large pedigrees in *ACTN2*^12^^,^^13^ and *MYOZ2*^14^ even though no excess of rare variants are observed in cases across these genes. However, even if these reported variants are disease-causing, other variants in these genes are expected to be very rarely associated with HCM, with the majority detected in cases likely to represent benign background variation. Confirming variant pathogenicity in such genes would require strong evidence of segregation in affected families, which is rarely available or pursued and precludes one of the main benefits of genetic testing—the predictive screening of family members with an undetermined phenotype. Clinical sequencing is therefore of very limited utility, as only specific well-characterized variants are actionable, with all other variants likely benign or uninterpretable. It is also notable that, at least in HCM, genes with a significant excess in cases account for the vast majority of known pathogenic and almost all clinically actionable variants.

In this study, we did not consider data on functional characterization as reliable evidence for a gene's involvement in HCM. Although cellular, tissue and animal model assays are valuable tools to evaluate the effects of genetic variants in established disease genes, there are several issues with using functional data to associate novel genes with HCM, including a lack of assay standardization and uncertainty about the translation of findings from laboratory to clinic. It is now apparent that some variants that produce clear HCM phenotypes in animal models are present at too high a population frequency to be fully penetrant pathogenic variants, e.g. *MYPN*:p.Y20C^16^ (ExAC allele count, AC = 111), *MYOZ2*:p.I246M^19^ (AC = 230), which limits the interpretability of such data. In our opinion, while model systems can provide valuable supporting information and mechanistic insight, only well-powered and statistically rigorous human studies can provide definitive evidence of a role in human disease.

Genes with a demonstrated functional effect for variants identified in HCM patients, but lacking in case excess or evidence of segregation in families, were highlighted as a distinct group in this study. Without this human genetic evidence, a primary pathogenic role for these genes cannot currently be justified and they will yield inconclusive results if included in genetic testing. It remains possible that, given their observed effect on *in vitro* and *in vivo* assays, rare variants in these genes may have some clinical role and could act to modify phenotypic expression in the presence of sarcomeric variants (as has been suggested for rare variants in the *TRIM63* and *TRIM54* genes^15^) However, studies on the effect on clinical phenotype of variants in these genes, in large family pedigrees or patient cohorts, would be needed to verify any such function.

This study used sequencing data from a variety of sources and platforms to compare variation in cases and the general population, with the differing sensitivities (particularly the exome sequencing of ExAC) a potential limitation of the analysis, though the ExAC frequencies were adjusted to account for variable coverage. Similarly, the combined case cohorts we used were derived from a variety of sources, including our data, clinical sequencing, and numerous published studies. While these would ideally be sourced from one study or laboratory to control for any technical confounding factors, maximizing the number of case samples is essential to accurately assess overall variation in highly constrained genes. Pending universal application of a comprehensive sequencing strategy to a single cohort of many thousands of patients, this type of meta-analysis provides the best opportunity for a thorough assessment of the role of genes in rare diseases like HCM.

Based on the characterization of the role of non-sarcomeric genes in HCM described in this study, we recommend that tests should currently be restricted to the eight core sarcomeric genes and *CSRP3*, *FHL1*, *FLNC*, *PLN*, *TNNC1*, as well as metabolic cardiomyopathy genes and possibly ACTN2 and MYOZ2 (though with the caveats on variant interpretation described above). The utility of genetic testing in HCM depends not only on the efficient identification of pathogenic variants but also reducing the uncertainty associated with detecting variants of unknown significance and the potential expense of investigating these, e.g. for samples sequenced as part of the 100 000 Genomes Project by Genomics England.

We have shown how candidate gene studies, if not properly controlled for the level of rare variation present at gene level in the population, can lead to erroneous genetic associations and demonstrated how datasets like ExAC can be used to overcome this. We also highlight the necessity for further evidence, particularly the segregation, or lack of it, of variants in affected families, to further clarify the role of the putative HCM genes described here. Resources like ClinVar,^20^ an archive of the clinical significance of human variation based on submissions by the medical genetics community, and ClinGen,^21^ which seeks to systematically evaluate the evidence for gene-disease relationships, will be critical in this regard. Finally, the findings underline the need for the discovery of additional genetic, epigenetic, and environmental causes of HCM that explain the large proportion of cases of unknown aetiology.

## Funding

National Institute for Health Research Biomedical Research Unit in Cardiovascular Disease at Royal Brompton & Harefield National Health Service Foundation Trust and Imperial College London; Wellcome Trust [107469/Z/15/Z, 090532/Z/09/Z]; Fondation Leducq [11 CVD-01]; Medical Research Council; British Heart Foundation [SP/10/10/28431]; British Heart Foundation Centre of Research Excellence in Oxford [RE/13/1/30181]; Oxford Biomedical Research Centre; National Medical Research Council (NMRC) Singapore [NMRC/STaR/0011/2012] and Health Innovation Challenge Fund funding from the Wellcome Trust and Department of Health, UK [HICF-R6-373].

This publication includes independent research commissioned by the Health Innovation Challenge Fund (HICF), a parallel funding partnership between the Department of Health and Wellcome Trust. The views expressed in this work are those of the authors and not necessarily those of the Department of Health or Wellcome Trust.

**Conflict of interest**: none declared. 

## Supplementary Material

Supplementary Notes

Supplementary Tables S1 S5 S7

Supplementary S2 S3 S4 S6
