# Supplementary material for: Defining the genetic architecture of hypertrophic cardiomyopathy: re-evaluating the role of non-sarcomeric genes
Source: Eur Heart J. 2017 Jan 11;38(46):3461–8. doi: 10.1093/eurheartj/ehw603 (PMC5837460; doi:10.1093/eurheartj/ehw603)
Supplement: Supplementary Notes [file ehw603_supplementary_notes.docx]

**Supplementary Note 1 - HCM disease terms from HGMD**

Cardiomyopathy, hypertrophic; Cardiomyopathy, hypertrophic with deafness; Cardiomyopathy, hypertrophic, with muscle hypertrophy; Cardiomyopathy, hypertropic; Hypertrophic cardiomyopathy; Hypertrophic cardiomyopathy, early onset; Hypertrophy, left ventricular; Increased left ventricular wall thickness; Left ventricular hypertrophy; Mitochondrial myopathy & hypertrophic cardiomyopathy; Nemaline myopathy and hypertrophic cardiomyopathy; Perinatal problems & hypertrophic cardiomyopathy; Proximal muscle weakness & hypertrophic cardiomyopathy; X-linked myopathy with hypertrophic cardiomyopathy

**Supplementary Note 2 – Sequencing details and bioinformatics variant calling pipeline**

Of the 804 HCM probands analysed as part of this study, 405 samples were sequenced using the Illumina TruSight Cardio Sequencing Kit^1^ (which includes 174 genes associated with inherited cardiac conditions (ICCs)) on the Illumina MiSeq and NextSeq platforms and 399 samples were sequenced using a custom Agilent SureSelect panel of genes associated with ICCs on the Life Technologies SOLiD 5500xl platform. Targeted DNA libraries were prepared according to manufacturers’ protocols before performing paired end sequencing.

*Illumina sequencing data*

Demultiplexing of sequence data were performed with MiSeq/NexSeq Control software or Bcl2FastQ conversion^2,3^ and the FastQ files were subjected to quality control with the FastQC v.0.10.1^4^. After low quality reads (<20) were trimmed using PrinSeq v0.20.4^5^, reads were aligned to the HG19 reference genome using BWA v0.7.10^6^. Picard v1.115^7^ and GATK v3.2-2^8^ were used to mark duplicate reads and perform local realignment around indels and base quality score recalibration. Bases covered by at least 10 reads with a mapping quality ≥10 and base quality ≥20 were denoted as “callable”, i.e. adequately covered for variant calling with recommended GATK parameters. Variant calling was performed with GATK HaplotypeCaller and UnifiedGenotyper.

*SOLiD sequencing data*

The SOLiD reads were aligned in colour space using LifeScope™ v2.5.1 “Targeted re-sequencing” pipeline^9^. The SOLiD Accuracy Enhancement Tool (SAET) was used to improve color call accuracy prior to mapping. Variants were called by the diBayes and SmallIndel packages in LifeScope software as well as GATK UnifiedGenotyper. Bases covered by at least 10 reads with a mapping quality ≥10 and base quality ≥20 were denoted as “callable”.

All detected variants from both platforms were functionally annotated using the Ensembl API v75_37^10^.

**References**

1. Pua CJ, Bhalshankar J, Miao K, Walsh R, John S, Lim SQ, Chow K, Buchan R, Soh BY, Lio PM, Lim J, Schafer S, Lim JQ, Tan P, Whiffin N, Barton PJ, Ware JS, Cook SA. Development of a Comprehensive Sequencing Assay for Inherited Cardiac Condition Genes. *J Cardiovasc Transl Res* 2016;

2. http://support.illumina.com/sequencing/sequencing_instruments/miseq/downloads.ilmn.

3. http://support.illumina.com/sequencing/sequencing_software/miseq_reporter/downloads.ilmn.

4. http://www.bioinformatics.babraham.ac.uk/projects/fastqc/.

5. Schmieder R, Edwards R. Quality control and preprocessing of metagenomic datasets. *Bioinformatics* 2011;**27**:863–864.

6. Li H, Durbin R. Fast and accurate long-read alignment with Burrows-Wheeler transform. *Bioinformatics* 2010;**26**:589–595.

7. http://picard.sourceforge.net.

8. McKenna A, Hanna M, Banks E, Sivachenko A, Cibulskis K, Kernytsky A, Garimella K, Altshuler D, Gabriel S, Daly M, DePristo MA. The Genome Analysis Toolkit: a MapReduce framework for analyzing next-generation DNA sequencing data. *Genome Res* 2010;**20**:1297–1303.

9. http://www.lifetechnologies.com/us/en/home/technical-resources/software-downloads/lifescope-genomic-analysis-software.html.

10. McLaren W, Pritchard B, Rios D, Chen Y, Flicek P, Cunningham F. Deriving the consequences of genomic variants with the Ensembl API and SNP Effect Predictor. *Bioinformatics* 2010;**26**:2069–2070.
