# Supplementary material for: Defining the genetic architecture of hypertrophic cardiomyopathy: re-evaluating the role of non-sarcomeric genes
Source: Eur Heart J. 2017 Jan 11;38(46):3461–8. doi: 10.1093/eurheartj/ehw603 (PMC5837460; doi:10.1093/eurheartj/ehw603)
Supplement: Supplementary S2 S3 S4 S6 [file ehw603_supplementary_tables_s2_s3_s4_s6.docx]

**Supplementary Tables S2, S3, S4, S6**

| **Validated Genes** | 1. | a) Significant excess in case cohorts over reference population AND  b) Maximum LOD score >3 |
| --- | --- | --- |
|  | | |
| **Strong Evidence** | 1. | a) No significant excess in case cohorts over reference population AND  b) Maximum LOD score >3. |
|  | 2. | a) Significant excess in case cohorts over reference population AND  b) Maximum LOD score >2 OR De novo variant OR Significant excess of individual variant in cases over reference population. |
| **Moderate Evidence** | 1. | a) No significant excess in case cohorts over reference population AND  b) Maximum LOD score >2 and <3 OR De novo variant OR Significant excess of individual variant in cases over reference population. |
|  | 2. | a) Significant excess in case cohorts over reference population AND  b) Maximum LOD score >1 and <2. |
| **Weak Evidence** | 1. | a) Significant excess in case cohorts over reference population AND  b) No maximum LOD score >1. |
|  | 2. | a) No significant excess in case cohorts over reference population AND  b) Maximum LOD score >1 and <2. |
|  | | |
| **Functional data only** | 1. | Variants demonstrated to have a functional effect in the absence of segregation data and without a significant excess in case cohorts over population/controls. |
|  | | |
| **No Evidence** | 1. | a) Reported variants are present at a frequency (MAF>0.0001 in ExAC) which is incompatible with being a causative HCM mutation AND  b) No evidence for family segregation, functional effects of variants or excess of variation in case cohorts. |
|  | 2. | a) No significant excess of rare variants in case cohorts over reference population AND  b) No evidence for family segregation or functional effects of variants. |

*Table S2: Summary of the criteria for the classification of putative HCM genes used in this study. A "significant excess" refers to combined HCM cohorts versus ExAC population data as described in Methods. Segregation data and functional evidence are derived from published reports.*

| **Category** | **Gene** | **Condition** | **Inheritance** | | **Refs** |
| --- | --- | --- | --- | --- | --- |
| **Rare conditions incl. LVH** | ACTA1 | Infantile-onset nemaline myopathy and HCM | De novo | | ^1,2^ |
|  | COX15 | Fatal infantile HCM and COX deficiency | Autosomal recessive | | ^3^ |
|  | MRPL3 | HCM with psychomotor retardation and multiple respiratory chain deficiency | Autosomal recessive | | ^4^ |
|  | MYO6 | HCM and sensorineural hearing loss | Autosomal dominant | | ^5^ |
|  | SLC25A4 | Complex phenotype including HCM, mild myopathy, lactic acidosis, ophthalmoplegia and exercise intolerance | Autosomal recessive | | ^6–9^ |
|  | | | | | |
| **Dual HCM / arrhythmia** | CACNA1C | HCM and Timothy/Brugada syndromes | - | ^10,11^ | |
|  | KCNQ1 | HCM and Long QT syndrome | - | ^10,12^ | |
|  | | | | | |
| **Phenocopies** | LAMP2 | Danon disease / Glycogen storage disease 2b | - | - | |
|  | GAA | Pompe disease / Glycogen storage disease 2 | - | - | |
|  | GLA | Fabry disease | - | - | |
|  | PRKAG2 | Wolff Parkinson White syndrome | - | - | |
|  | TTR | Hereditary transthyretin-related amyloidosis | - | - | |

*Table S3: Summary of genes associated with complex phenotypes that include left ventricular hypertrophy.*

| **Gene** | **Year (ref)** | **HGMD variants** | **HGMD references** | **ExAC freq.** | **Clinical freq.** | **Case excess** | **Path. variants** |
| --- | --- | --- | --- | --- | --- | --- | --- |
| MYH7 | 1989^13^ | 424 | 168 | 1.40% | 14.22% | 12.82%* | 597 |
| MYBPC3 | 1990^14^ | 505 | 155 | 1.97% | 19.03% | 17.06%* | 932 |
| TNNT2 | 1994^15^ | 65 | 75 | 0.24% | 1.95% | 1.71%* | 73 |
| TPM1 | 1994^15^ | 22 | 36 | 0.09% | 1.48% | 1.40%* | 31 |
| MYL2 | 1996^16^ | 25 | 29 | 0.18% | 1.10% | 0.92%* | 30 |
| MYL3 | 1996^16^ | 23 | 20 | 0.18% | 0.88% | 0.70%* | 9 |
| TNNI3 | 1997^17^ | 52 | 48 | 0.23% | 2.23% | 2.01%* | 89 |
| ACTC1 | 1999^18^ | 17 | 16 | 0.06% | 0.53% | 0.46%* | 7 |

*Table S4: Overview of data demonstrating the role of eight validated sarcomeric genes in HCM. Displayed are the year of the initial report, the distinct number of variants and references in HGMD (version 2015.3) that associate the gene with HCM, the frequency of rare variants in ExAC and clinical genetics cohorts (using combined data from OMGL and LMM laboratories), the case excess of rare variants in the clinical cohorts over ExAC (* = Fisher’s exact test p-value < 0.0001) and the total number of cases with clinical-grade pathogenic and likely pathogenic variants from the OMGL and LMM cohorts.*

| **Demographic / phenotype** | **Total (781)** | **Genotype status** | | | **p-values** | | |
| --- | --- | --- | --- | --- | --- | --- | --- |
|  |  | **Valid HCM gene (254)** | **Other gene (89)** | **Negative (438)** | **valid vs neg.** | **valid vs other** | **neg. vs other** |
| **Gender (% male)** | 575 (73.6%) | 176 (69.3%) | 71 (79.8%) | 328 (74.9%) | 0.112 | 0.074 | 0.416 |
| **FH (HCM)** | 147 (23.7%) | 83 (38.8%) | 12 (17.9%) | 52 (15.3%) | **<0.0001** | **0.0017** | 0.585 |
| **FH (SCD)** | 141 (24.2%) | 64 (33.3%) | 9 (13.6%) | 68 (21.0%) | **0.0024** | **0.0024** | 0.234 |
| **Age** | 56.1±15.0 | 50.9±14.9 | 58.6±15.3 | 58.5±14.3 | **<0.0001** | **<0.0001** | 0.997 |
| **Max LVWT (mm)** | 19.05±4.43 | 19.76±4.70 | 18.25±3.56 | 18.81±4.39 | **0.019** | **0.22** | 0.540 |

*Table S6: Demographic and clinical characteristics of HCM patients sequenced in this study by genotype status. Valid HCM gene group contains rare variants (ExAC frequency < 1x10^-4^) in the following genes – MYH7, MYBPC3, TNNT2, TNNI3, TPM1, MYL2, MYL3, ACTC1, CSRP3, FHL1, PLN, TNNC1. Other gene group contains rare variants (ExAC frequency < 1x10^-4^) in the following genes – ACTN2, ANKRD1, CALR3, CASQ2, CAV3, CRYAB, FHL2, FXN, JPH2, KLF10, LDB3, LMNA, MYH6, MYLK2, MYOM1, MYOZ2, MYPN, NEXN, OBSCN, PDLIM3, SRI, TCAP, TRIM54, TRIM55, TRIM63, VCL – but not rare variants in valid HCM genes. Negative indicates patients without rare variants in any of these genes. P-values are from Fisher’s exact test (for Gender and FH) or Analysis of Variance test (ANOVA) followed by Tukey’s multiple comparison tests (for age and max LVWT).*

**REFERENCES**

1. D’Amico A, Graziano C, Pacileo G, Petrini S, Nowak KJ, Boldrini R, Jacques A, Feng J-J, Porfirio B, Sewry CA, Santorelli FM, Limongelli G, Bertini E, Laing N, Marston SB. Fatal hypertrophic cardiomyopathy and nemaline myopathy associated with ACTA1 K336E mutation. *Neuromuscul Disord* 2006;**16**:548–552.

2. Kim S-Y, Park Y-E, Kim H-S, Lee C-H, Yang DH, Kim D-S. Nemaline myopathy and non-fatal hypertrophic cardiomyopathy caused by a novel ACTA1 E239K mutation. *J Neurol Sci* 2011;**307**:171–173.

3. Antonicka H, Mattman A, Carlson CG, Glerum DM, Hoffbuhr KC, Leary SC, Kennaway NG, Shoubridge EA. Mutations in COX15 produce a defect in the mitochondrial heme biosynthetic pathway, causing early-onset fatal hypertrophic cardiomyopathy. *Am J Hum Genet* 2003;**72**:101–114.

4. Galmiche L, Serre V, Beinat M, Assouline Z, Lebre A-S, Chretien D, Nietschke P, Benes V, Boddaert N, Sidi D, Brunelle F, Rio M, Munnich A, Rötig A. Exome sequencing identifies MRPL3 mutation in mitochondrial cardiomyopathy. *Hum Mutat* 2011;**32**:1225–1231.

5. Mohiddin SA, Ahmed ZM, Griffith AJ, Tripodi D, Friedman TB, Fananapazir L, Morell RJ. Novel association of hypertrophic cardiomyopathy, sensorineural deafness, and a mutation in unconventional myosin VI (MYO6). *J Med Genet* 2004;**41**:309–314.

6. Palmieri L, Alberio S, Pisano I, Lodi T, Meznaric-Petrusa M, Zidar J, Santoro A, Scarcia P, Fontanesi F, Lamantea E, Ferrero I, Zeviani M. Complete loss-of-function of the heart/muscle-specific adenine nucleotide translocator is associated with mitochondrial myopathy and cardiomyopathy. *Hum Mol Genet* 2005;**14**:3079–3088.

7. Echaniz-Laguna A, Chassagne M, Ceresuela J, Rouvet I, Padet S, Acquaviva C, Nataf S, Vinzio S, Bozon D, Mousson de Camaret B. Complete loss of expression of the ANT1 gene causing cardiomyopathy and myopathy. *J Med Genet* 2012;**49**:146–150.

8. Strauss KA, DuBiner L, Simon M, Zaragoza M, Sengupta PP, Li P, Narula N, Dreike S, Platt J, Procaccio V, Ortiz-González XR, Puffenberger EG, Kelley RI, Morton DH, Narula J, Wallace DC. Severity of cardiomyopathy associated with adenine nucleotide translocator-1 deficiency correlates with mtDNA haplogroup. *Proc Natl Acad Sci U S A* 2013;**110**:3453–3458.

9. Körver-Keularts IMLW, Visser M de, Bakker HD, Wanders RJA, Vansenne F, Scholte HR, Dorland L, Nicolaes GAF, Spaapen LMJ, Smeets HJM, Hendrickx ATM, Bosch BJC van den. Two Novel Mutations in the SLC25A4 Gene in a Patient with Mitochondrial Myopathy. *JIMD Rep* 2015;**22**:39–45.

10. D’Argenio V, Frisso G, Precone V, Boccia A, Fienga A, Pacileo G, Limongelli G, Paolella G, Calabrò R, Salvatore F. DNA sequence capture and next-generation sequencing for the molecular diagnosis of genetic cardiomyopathies. *J Mol Diagn* 2014;**16**:32–44.

11. Boczek NJ, Ye D, Jin F, Tester DJ, Huseby A, Bos JM, Johnson AJ, Kanter R, Ackerman MJ. Identification and Functional Characterization of a Novel CACNA1C-Mediated Cardiac Disorder Characterized by Prolonged QT Intervals With Hypertrophic Cardiomyopathy, Congenital Heart Defects, and Sudden Cardiac Death. *Circ Arrhythm Electrophysiol* 2015;**8**:1122–1132.

12. Wang L, Zuo L, Hu J, Shao H, Lei C, Qi W, Liu Y, Miao Y, Ma X, Huang CL-H, Wang B, Zhou X, Zhang Y, Liu L. Dual LQT1 and HCM phenotypes associated with tetrad heterozygous mutations in KCNQ1, MYH7, MYLK2, and TMEM70 genes in a three-generation Chinese family. *Europace* 2015;

13. Jarcho JA, McKenna W, Pare JA, Solomon SD, Holcombe RF, Dickie S, Levi T, Donis-Keller H, Seidman JG, Seidman CE. Mapping a gene for familial hypertrophic cardiomyopathy to chromosome 14q1. *N Engl J Med* 1989;**321**:1372–1378.

14. Solomon SD, Jarcho JA, McKenna W, Geisterfer-Lowrance A, Germain R, Salerni R, Seidman JG, Seidman CE. Familial hypertrophic cardiomyopathy is a genetically heterogeneous disease. *J Clin Invest* 1990;**86**:993–999.

15. Thierfelder L, Watkins H, MacRae C, Lamas R, McKenna W, Vosberg HP, Seidman JG, Seidman CE. Alpha-tropomyosin and cardiac troponin T mutations cause familial hypertrophic cardiomyopathy: a disease of the sarcomere. *Cell* 1994;**77**:701–712.

16. Poetter K, Jiang H, Hassanzadeh S, Master SR, Chang A, Dalakas MC, Rayment I, Sellers JR, Fananapazir L, Epstein ND. Mutations in either the essential or regulatory light chains of myosin are associated with a rare myopathy in human heart and skeletal muscle. *Nat Genet* 1996;**13**:63–69.

17. Kimura A, Harada H, Park JE, Nishi H, Satoh M, Takahashi M, Hiroi S, Sasaoka T, Ohbuchi N, Nakamura T, Koyanagi T, Hwang TH, Choo JA, Chung KS, Hasegawa A, Nagai R, Okazaki O, Nakamura H, Matsuzaki M, Sakamoto T, Toshima H, Koga Y, Imaizumi T, Sasazuki T. Mutations in the cardiac troponin I gene associated with hypertrophic cardiomyopathy. *Nat Genet* 1997;**16**:379–382.

18. Mogensen J, Klausen IC, Pedersen AK, Egeblad H, Bross P, Kruse TA, Gregersen N, Hansen PS, Baandrup U, Borglum AD. Alpha-cardiac actin is a novel disease gene in familial hypertrophic cardiomyopathy. *J Clin Invest* 1999;**103**:R39–R43.
